# Drawing human pedigree charts with DrawPed

**DOI:** 10.1093/nar/gkae336

**Published:** 2024-05-10

**Authors:** Janina Schönberger, Robin Steinhaus, Dominik Seelow

**Affiliations:** Exploratory Diagnostic Sciences, Berliner Institut für Gesundheitsforschung@Charité, Charitéplatz 1, 10117, Berlin, Germany; Institut für medizinische Genetik und Humangenetik, Charité – Universitätsmedizin Berlin, Augustenburger Platz 1, 13353, Berlin, Germany; Exploratory Diagnostic Sciences, Berliner Institut für Gesundheitsforschung@Charité, Charitéplatz 1, 10117, Berlin, Germany; Institut für medizinische Genetik und Humangenetik, Charité – Universitätsmedizin Berlin, Augustenburger Platz 1, 13353, Berlin, Germany; Exploratory Diagnostic Sciences, Berliner Institut für Gesundheitsforschung@Charité, Charitéplatz 1, 10117, Berlin, Germany; Institut für medizinische Genetik und Humangenetik, Charité – Universitätsmedizin Berlin, Augustenburger Platz 1, 13353, Berlin, Germany

## Abstract

Creating pedigree charts is a recurring task in biomedical research, but there are few online tools for drawing complex human pedigrees available and even fewer are free. With DrawPed we aim to close this gap. DrawPed automatically draws pedigree charts from standard PED format pedigree files. Users can also create pedigrees from scratch and interactively edit existing pedigrees. The application can display conditions not captured in a PED file such as deceased persons or suspected consanguinity of parents. Pedigree charts are displayed as SVGs, which are scalable and hence publication-ready. Pedigrees can be exported as PED files for storage, exchange, or use in other applications. DrawPed is open source and freely available at https://www.genecascade.org/DrawPed/.

## Graphical Abstract

**Graphical Abstract:**
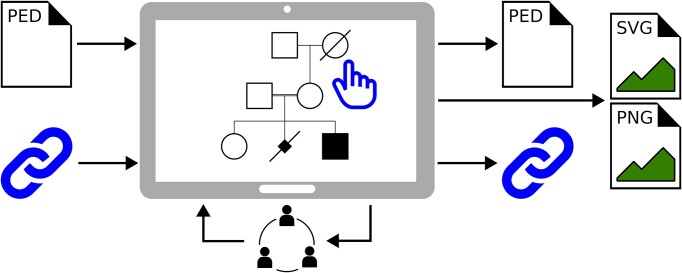

## Introduction

A pedigree chart is essential to conveying the structure of a family and determining the most likely mode of inheritance of a genetic disease. With the advent of high-throughput sequencing, the awareness for potentially genetic causes of disorders and the ability to analyse the segregation of DNA variants in affected family members have spread to other medical disciplines and biomedical research in general. In addition to studying clearly monogenic disorders, they are also a simple way of comparing phenotypic data with genetic information in diseases without a single genetic cause. Whilst pedigree charts have always been a cornerstone of human genetics, their relevance in other disciplines is growing as well.

Creating a pedigree chart according to the conventions (1) is however less simple than it may seem at first glance: male parents should be placed on the left whenever possible, children should be centred under their parents and there should not be any overlapping lines connecting parents, children or partners. The challenge of adhering to these standards becomes difficult to address when consanguinity leads to ‘loops’ in the chart or when parents have children with different partners. Due to this complexity, relatively few tools aimed at drawing such charts exist (e.g. HaploPainter (2), Madeline Pedigree Drawing Engine (3), HaploForge (4), pedigreejs (5) ped_draw (6), pedbuildr (7)), many of which are commercial (e.g. Cyrillic (8), Progeny Pedigree Builder, Visual Paradigm, smartdraw) and offer limited functionality in their free versions. Even fewer tools are web-based (see Table 1), although web-based applications offer significant benefits, such as the convenience of sharing content via direct links, access to the newest features through automatic updates, and the flexibility to use them on any device without the need for installation. These advantages are of particular importance in settings with installation restrictions, such as healthcare environments. However, many researchers and clinicians still use classical drawing applications which are already installed on their computers.

**Table 1.** Comparison with free pedigree chart drawing tools

Parameter	DrawPed	ped_draw (6)	pedigreejs (5)	HaploForge (4)	Madeline 2.0 PDE (3)	HaploPainter (2)	Progeny Pedigree Builder^a^
Father placed on the left by default	+	−	−	−	−	o	o
Automatic detection of consanguinity	+	−	+	−	+	+	−
Deceased persons	+	−	+	−	+	+	+
Multiple traits	−	−	+	−	+	o	+
Automatic pedigree chart from PED file	+	o	+	−	−	+	−
Export of PED files	+	o	−	+	−	+	−
Export of pedigree chart as image	+	+	+	−	+	+	+
Manual creation of pedigree chart	+	−	+	+	+	+	+
Interactive editing	+	−	+	+	o	+	+
Manually move individuals in the chart	−	−	−	+	−	+	+
Web version available	+	+	+	+	+	−	+
Programmatic access to website	+	−	+	−	−	−	−
Open source	+	+	+	+	+	+	−

Symbols: +: yes/fully implemented, o: partly implemented, −: no/not implemented/does not work. All tests were performed in February 2024. We tested the respective graphical (web) user interfaces, not command-line access. Please note that HaploPainter and Haploforge are mainly aimed at displaying haplotypes.

^a^ https://www.progenygenetics.com/online-pedigree/.

Whilst drawing pedigree charts is a non-trivial problem, capturing the structure of families is easy: the PED (or pre-makeped) format (9) has been used for decades. Here, each person is stored with their family ID, their own ID, IDs of father and mother, sex and the phenotype (usually affected or unaffected) in consecutive columns. This simple structure is sufficient to describe the relations within a family. It does not allow annotating further information such as miscarriages or deceased persons; information which can and should be shown in a pedigree chart. Many pedigree chart drawing tools hence use their own format to capture families (e.g. Madeline Pedigree Drawing Engine (3) or pedigreejs (5)). While this improves their drawing capabilities, it reduces the level of interoperability.

## DrawPed

As there is a high demand to use and display family structures in biomedical research and clinical genetics and to easily combine this with patient databases or downstream applications, we have developed DrawPed. It is web-based and can be used from computers as well as from mobile devices. It allows integration into existing pipelines and collaborative work on a pedigree.

DrawPed is based on the PED format so that pedigree information can easily be imported and exported from and to other applications such as PLINK (10) or linkage software (e.g. GENEHUNTER (11) or ALLEGRO (12)). Pedigrees can also be generated from scratch and interactively modified without the need to manually create a PED file. Since our application can only display one family, users are asked which family they want to edit, should they upload a multi-family PED file. Pedigrees can also be generated from scratch and interactively modified without the need to provide a PED file.

DrawPed is explicitly designed as a lightweight and simple solution for human families. It adheres to the universally accepted standard defined by Bennett *et al.* (1) and can display most relationships commonly found in human pedigrees, including consanguineous unions and individuals with two partners. It is not designed for drawing pedigrees typically found in animal breeding (e.g. backcrossings, see Limitations below). DrawPed will display error messages when it encounters situations it cannot handle or which are impossible, such as persons being their own ancestors.

**Figure 1.**
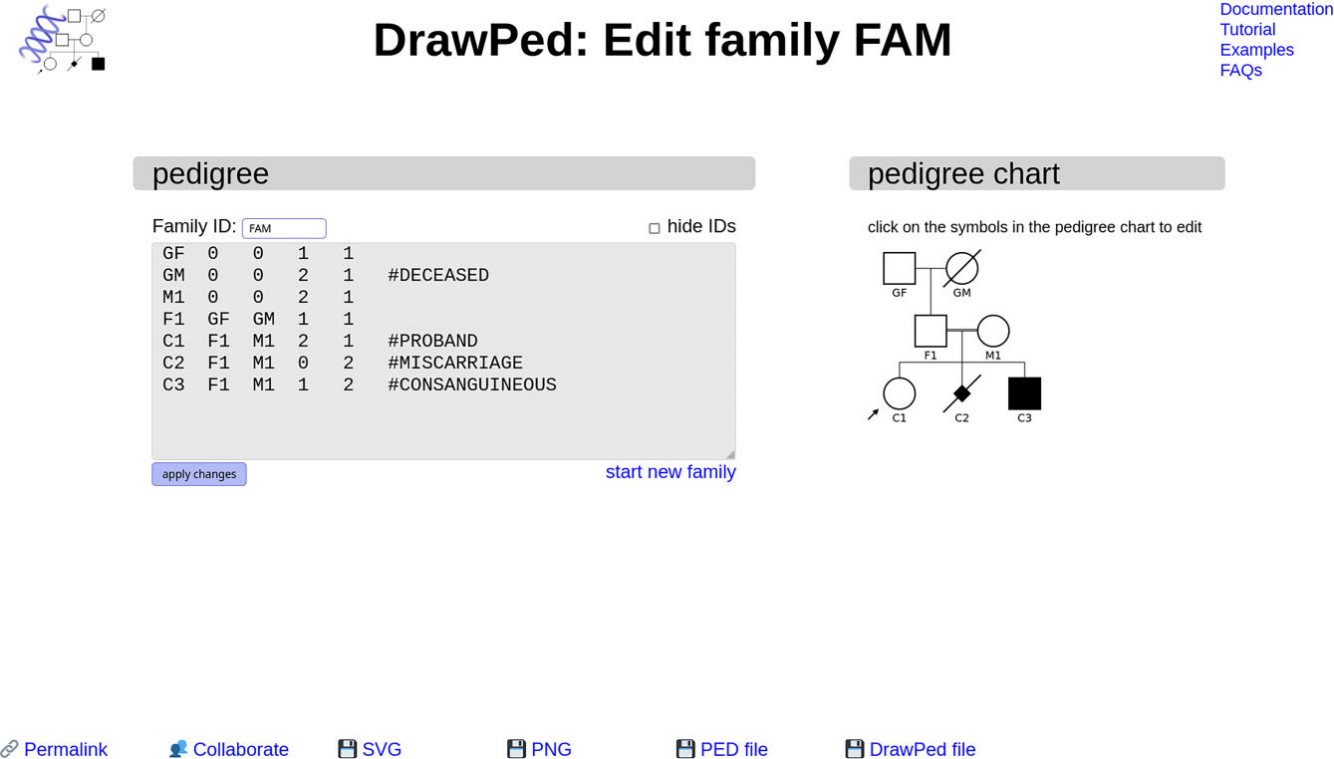
Interface of DrawPed. On the left, the pedigree data (PED file) is shown and can be edited. The family ID (which is of course the same for all family members, FAM in this example) is not included but displayed above the text box to reduce complexity. The pedigree chart is an interactive SVG graphic on the right, here with C1–C3 manually marked as children of consanguineous parents (depicted as a double line between their parents F1 & M1). Clicking on a person allows changing various parameters (e.g. phenotype) as well as adding family members or deleting the person. The buttons on the bottom provide a stable link to the pedigree (permalink), and the options to set up a collaborative project, to download the pedigree chart as an image in SVG and PNG format, and the corresponding PED file.

The software also shows information which is not part of the standard pedigree file format: DrawPed indicates deceased persons, miscarriages, and probands/consultands (1) (see Figure 1). This additional information is stored as comments in an additional column of the pedigree data, but DrawPed also allows exporting families in the standard PED format without this auxiliary information.

Consanguinity is automatically detected and indicated with a double line between the parents. If, as in many cases in human genetics, the exact structure of a consanguineous family is not known, users can define parents as being related. This will result in a double line connecting them, even if their own ancestors are not included in the pedigree chart.

## Interactive editing

DrawPed offers interactive editing of pedigree charts by clicking on the symbols in the pedigree. Options include adding family members, removing persons, changing sex, affection and ID, and adding auxiliary information (such as proband status). Based on the logic of the pedigree, some options are disabled, e.g. a miscarried person cannot have children and the sex of parents cannot be changed.

It is possible to build a pedigree from scratch without a pedigree file, interactively edit existing pedigree files or start with pedigree templates (we provide some on our website). This enables the user to easily obtain logically consistent pedigree files without having to manually create them, as a PED file is generated from the input and displayed in a text box (Figure 1). Manually editing the PED file in the graphical user interface is of course also possible.

## Exporting and sharing

Pedigree data can be stored and shared as hyperlinks which are automatically created when drawing a pedigree chart. This static hyperlink (‘permalink’) can easily be shared and will open DrawPed with exactly the same pedigree structure and chart.

DrawPed also offers a collaboration mode that allows users to work on the same pedigree together. The pedigree is stored on our server for one week and can be edited by all collaborators.

Pedigree charts can be exported as Scalable Vector Graphics (SVG) or as raster images (Portable Network Graphics, PNG). Whilst IDs of the different family members are required to build the family structure, they can be omitted in the chart for simplicity.

We provide extensive documentation and an illustrative tutorial, along with templates and examples, on our website.

### Algorithm

DrawPed's algorithm consists of two main phases: indexing and drawing. In the indexing phase, the algorithm performs a depth-first traversal of the chart, assigning a generation number and an index to each node in the pedigree. This index specifies the person's position within its generation for the subsequent drawing phase. For complex families, the algorithm may revisit nodes multiple times until final indexes are found.

In the drawing phase, the algorithm identifies nuclear families (parents and children) and determines the required horizontal distance of the parents in the chart so that their ancestors can be drawn above them.

In the next step, children are centred below their parents. Depending on the pedigree, this may either require moving the parents or the children; we therefore iteratively adjust the positions until an optimal placement in the canvas is found and there is no overlap of symbols or lines.

### Programmatic access

DrawPed can easily be used from other applications: Using an HTTPS POST request, a PED file can be opened in the web interface and the chart will be created automatically. Pedigree information can also be transmitted as hyperlinks, which store the complete pedigree information (including additional annotations) in a compressed format. Last but not least, DrawPed can be used to return a pedigree chart without opening the web interface at all, providing a simple way to create pedigree charts from existing PED files. We provide a Perl script for automatically generating multiple pedigree charts from a multi-family PED file on our website.

Examples for the different ways to access DrawPed from other applications are given in the documentation.

### Limitations

DrawPed is aimed at and restricted to ‘common’ human pedigrees. There are several cases it cannot handle such as different traits in the same family, nested consanguineous ‘loops’ (where pedigrees cannot be drawn without overlapping lines between parents and children), generation-spanning parents, backcrossing, or large ancestry tables.

The position of persons within the pedigree chart is determined automatically according to the conventions (1) and with the persons sorted by their IDs and cannot be changed manually. Depending on the use case, we recommend to either use graph drawing applications, pedigree software that allows to move persons (e.g. HaploPainter, which is however not web-based), or even classical drawing tools.

A detailed list of limitations along with suggestions to circumvent these is given in the documentation and the FAQs on our website.

### Implementation and availability

DrawPed is open source and completely free to use without registration at https://www.genecascade.org/DrawPed/.

DrawPed is programmed in Perl. We use plain JavaScript (without external libraries) for interactive features such as adding or editing persons. Pedigrees are not stored on our server (except for collaboration projects) and DrawPed does not connect to any other server. The application does not require a web server to create pedigree charts from pedigree files and can easily be used locally. The code is available from https://git-ext.charite.de/genecascade/drawped/ and licensed under CC BY-SA 4.0.

### Comparison with other applications

Table 1 shows a feature comparison of DrawPed with other applications that are capable of drawing human pedigrees. Tools that require registration or payment or do no longer work are not included. DrawPed seems to be the only free web-based tool with working PED file import and export. Several pedigree drawing tools we tested do not adhere to the current standards (1) or do not alert the user of logical inconsistencies in the pedigree. An extended version of Table 1 including further aspects of their respective chart drawing capabilities, usability, interoperability, interactive features, and technical considerations is available in the documentation on our website (https://www.genecascade.org/DrawPed/comparison.html).

Building on our tests of other applications, we tried to make DrawPed as intuitive and robust as possible and to provide helpful error messages in case of problems.

## Outlook

We will integrate DrawPed into several of our web-based applications (https://www.genecascade.org/) to make access to family data more comfortable.

## Data Availability

DrawPed is open source and completely free to use without registration at https://www.genecascade.org/DrawPed/.
